# First-Principles Study of Mechanical and Thermodynamic Properties of Binary and Ternary CoX (X = W and Mo) Intermetallic Compounds

**DOI:** 10.3390/ma14061404

**Published:** 2021-03-13

**Authors:** Yunfei Yang, Changhao Wang, Junhao Sun, Shilei Li, Wei Liu, Hao Wu, Jinshu Wang

**Affiliations:** Key Laboratory of Advanced Functional Materials, Education Ministry of China, Faculty of Materials and Manufacturing, Beijing University of Technology, Beijing 100124, China; yangyunfei@emails.bjut.edu.cn (Y.Y.); sunjunhao@emails.bjut.cn (J.S.); lisl511@emails.bjut.edu.cn (S.L.); liuw@bjut.edu.cn (W.L.); wh020033@163.com (H.W.)

**Keywords:** first-principles calculations, cobalt, molybdenum, tungsten, alloys, mechanical property, thermodynamic property

## Abstract

In this study, the structural, elastic, and thermodynamic properties of DO_19_ and L1_2_ structured Co_3_X (X = W, Mo or both W and Mo) and μ structured Co_7_X_6_ were investigated using the density functional theory implemented in the pseudo-potential plane wave. The obtained lattice constants were observed to be in good agreement with the available experimental data. With respect to the calculated mechanical properties and Poisson’s ratio, the DO_19_-Co_3_X, L1_2_-Co_3_X, and μ-Co_7_X_6_ compounds were noted to be mechanically stable and possessed an optimal ductile behavior; however, L1_2_-Co_3_X exhibited higher strength and brittleness than DO_19_-Co_3_X. Moreover, the quasi-harmonic Debye–Grüneisen approach was confirmed to be valid in describing the temperature-dependent thermodynamic properties of the Co_3_X and Co_7_X_6_ compounds, including heat capacity, vibrational entropy, and Gibbs free energy. Based on the calculated Gibbs free energy of DO_19-_Co_3_X and L1_2_-Co_7_X_6_, the phase transformation temperatures for DO_19-_Co_3_X to L1_2_-Co_7_X_6_ were determined and obtained values were noted to match well with the experiment results.

## 1. Introduction

Cobalt (Co)-based superalloys are suitable candidates for modern aircraft engines, turbo superchargers, and chemical industrial materials requiring optimal performance at elevated temperatures. This is owing to their prominent creep resistance during long-term high-temperature exposure, which is guaranteed by the addition of the various refractory elements, such as Cr, Mo, W and Re [1,2,3,4,5,6,7]. Hence, the incorporation of W and Mo into Co has attracted widespread research attention [8]. In addition, Co–W and Co–Mo coatings are considered to be an appropriate alternative to replace Cr coatings, due to the superior mechanical, tribological, and corrosion resistance properties of these alloys [9,10,11]. Moreover, cobalt still retains the magnetic properties in cases where the tungsten and molybdenum concentrations in the alloy are less than 20%. Therefore, the Co–W and Co–Mo alloys can be utilized in disk and magnetic film memory devices [12]. 

Due to the similar characteristics of W and Mo, the Co–W and Co–Mo binary alloys have close physical and chemical properties, which means that they can be thought of as a substitute for each other in many cases. Due to the differences of W and Mo in melting points, density etc., the performance gap between binary alloys of Co–W and Co–Mo also cannot be negligible in specific applications. However, the insufficient studies on Co–W–Mo ternary alloys make it inconclusive as to whether ternary alloys can conform to the performance of binary Co–W/Mo alloys. Therefore, it is meaningful to study the difference on properties of binary and ternary alloys of Co–W/Mo for achieving continuous adjustment of alloy performances.

The binary and ternary alloy phases of Co–W/Mo have been explored by many studies. Based on the experimental constituent binary phase diagrams of the Co–W system [13], the highly stable geometrically close-packed (GCP) phase (Co_3_X) and topological close-packed (TCP) μ phase (Co_7_X_6_) have been confirmed in the alloys, which is also similar in the Co–Mo system. In addition, the σ phase (Co_2_X_3_) in Co–Mo alloys has also been verified to be stable [14]. Among the GCP phases of the Co–W or Co–Mo systems, Co_3_X (X = Mo and W) in the DO_19_ structures, belonging to the P6_3_/mmc space group, has been widely accepted as the low temperature phase in the alloys with a high content of Co. Meanwhile, the GCP L1_2_ phase Co_3_W has been identified in the space group of Fm-3m by Sato et al. in the Co–W and Co–W–Al alloys. This has attracted a significant attention due to the effective strengthening of the Co-based high temperature materials, similar to Ni-based superalloys strengthened by the γ’-Ni_3_Al precipitates with an L1_2_ crystal structure [6]. A few studies in the literature have also focused on the Co–Mo–W ternary system [15,16]. Ishchenko et al. reported that the σ (Co_2_Mo_3_) phase was not identified in the isothermal section at 1000 °C, which implied that the solubility of the σ (Co_2_Mo_3_) phase in the Co–Mo–W ternary system was possibly small. Due to the isostructural features of Co_3_W/Mo and Co_7_W/Mo_6_, Co_7_(W, Mo)_6_ has been observed to form the completely continuous solid solution phases in the alloy. Ren et al. have studied the alloying effect of tungsten on the µ phase of Co_7_Mo_6_ by employing the first-principles calculations. The authors observed that the addition of tungsten promoted the stability of the μ phase in Co_7_Mo_6_, and W tended to participate in the formation of the phase [17]. However, the mechanical and thermodynamic properties of the Co–W/Mo systems needed to be explored further.

In the past few years, the theoretical calculations based on the density functional theory (DFT) have been employed to reveal and predict the structural, mechanical, and physical properties of the Co-based alloys. In a related study, Xu et al. [18] computed the electronic band structure as well as mechanical and thermal dynamic properties of Co_3_X (X = Ti, Ta, W, V and Al) with the cubic L1_2_ and DO_19_ phases. The authors confirmed that the DO_19_ phase Co_3_W existed as a stable phase. Arikan et al. [19] studied the structural, electronic, elastic, and dynamic properties of Co_3_W in the L1_2_ phase. Likewise, Yuan et al. [20] studied the magnetic properties of Co_3_W by employing the first-principles calculations. Li et al. conducted first-principles calculations of the site occupancy and electronic properties of Co_7_W_6_ doped with Re [21]. The authors reported that the stability of Co_7_W_6_ enhanced after Re doping, and Re was prone to the formation of the Co_7_W_6_ μ phase. However, the calculations of the Co–W–Mo system are still limited.

In the current study, the DO_19_, L1_2_, and μ structures of binary Co–W and Co–Mo, as well as ternary Co–W–Mo systems have been studied by employing first-principles calculations. The elastic stiffness matrix *C_ij_* and elastic compliance matrix *S_ij_* parameters have been calculated, and the values of the bulk (*B*), shear (*G*) and Young’s (*E*) moduli as well as Poisson’s ratio (σ) were subsequently derived. Based on the mechanical parameters, the brittleness and plasticity of the compounds have been discussed. In addition, heat of formation, cohesive energy, heat capacity and Gibbs free energy have also been calculated.

## 2. Computational Method

In the current study, the first-principles calculations have been performed by using the plane wave basis projector augmented wave (PAW) method [22,23] in the DFT framework, as implemented in the Vienna Ab initio Simulation Package (VASP) code [24,25,26]. The local density is described through the generalized gradient approximation (GGA) of Perdew–Burke–Ernzerhof (PBE) [27]. A plane wave basis set energy cut-off of 500 eV was applied during the calculations to ensure accuracy. Brillouin zone sampling was performed by using the gamma point centered k-meshes 6 × 6 × 8, 9 × 9 × 9, and 7 × 7 × 1 for the DO_19_, L1_2_ and μ structures, respectively. Throughout the calculations, the convergence of the total energy and maximum force of the ionic relaxation were set to be less than 10^−6^ eV and 10^−2^ eV/Å, respectively.

The second order elastic constants were calculated using the efficient stress–strain energy method [28,29]. Accordingly, the stresses and strains satisfied Hooke’s law for small deformations, as per Equation (1):

(1) $$\left(\begin{array}{c} \sigma_{1}\\ \sigma_{2}\\ \sigma_{3}\\ \sigma_{4}\\ \sigma_{5}\\ \sigma_{6} \end{array}\right)=\left(\begin{array}{ccc} \begin{array}{c} \begin{array}{cc} C_{11} & \;C_{12} \end{array}\\ \begin{array}{cc} C_{21} & \;C_{22} \end{array} \end{array} & \begin{array}{c} \begin{array}{cc} C_{13} & \;C_{14} \end{array}\\ \begin{array}{cc} C_{23} & \;C_{24} \end{array} \end{array} & \begin{array}{c} \begin{array}{cc} C_{15} & \;C_{16} \end{array}\\ \begin{array}{cc} C_{25} & \;C_{26} \end{array} \end{array}\\ \begin{array}{c} \begin{array}{cc} C_{31} & \;C_{32} \end{array}\\ \begin{array}{cc} C_{41} & \;C_{42} \end{array} \end{array} & \begin{array}{c} \begin{array}{cc} C_{33} & \;C_{34} \end{array}\\ \begin{array}{cc} C_{43} & \;C_{44} \end{array} \end{array} & \begin{array}{c} \begin{array}{cc} C_{35} & \;C_{36} \end{array}\\ \begin{array}{cc} C_{45} & \;C_{46} \end{array} \end{array}\\ \begin{array}{c} \begin{array}{cc} C_{51} & \;C_{52} \end{array}\\ \begin{array}{cc} C_{61} & \;C_{62} \end{array} \end{array} & \begin{array}{c} \begin{array}{cc} C_{53} & \;C_{54} \end{array}\\ \begin{array}{cc} C_{63} & \;C_{64} \end{array} \end{array} & \begin{array}{c} \begin{array}{cc} C_{55} & \;C_{56} \end{array}\\ \begin{array}{cc} C_{65} & \;C_{66} \end{array} \end{array} \end{array}\right)\left(\begin{array}{c} e_{1}\\ e_{2}\\ e_{3}\\ e_{4}\\ e_{5}\\ e_{6} \end{array}\right)$$

where *σ_i_*, *C_ij_* and *e_i_* are the stress vector, second order elastic constants and strain vector, respectively. The Taylor expansion of the internal energy of the deformed crystal under the micro-strain component yields can be expressed as:

(2) $$E\left(V,e\right)=E\left(V_{0},0\right)+\frac{V_{0}}{2}\;\overset{6}{\underset{i,j=1}{\sum }}C_{ij}e_{i}e_{j}$$

where *V*_0_ and *E*(*V*_0_,0) are the equilibrium volume and energy of the undistorted structure, respectively. In the strain set (*e* = *e*_1_, *e*_2_, *e*_3_, *e*_4_, *e*_5_ and *e*_6_), *e*_1_, *e*_2_ and *e*_3_ refer to the normal strains, and *e*_4_, *e*_5_ and *e*_6_ indicate the shear strains. These are used to generate the small deformations of the unit cell. The crystalline lattice vectors before (R) and after (R’) the deformation are related as follows:

(3) $$R\prime =R\cdot \left(\begin{array}{ccc} e_{1}+1 & \frac{e_{6}}{2} & \frac{e_{5}}{2}\\ \frac{e_{6}}{2} & e_{2}+1 & \frac{e_{4}}{2}\\ \frac{e_{5}}{2} & \frac{e_{4}}{2} & e_{3}+1 \end{array}\right)$$

The elastic constants can be obtained by fitting the energy versus strain curve with the quadratic polynomial function.

## 3. Results and Discussion

### 3.1. Geometry and Structural Properties

**Figure 1:**
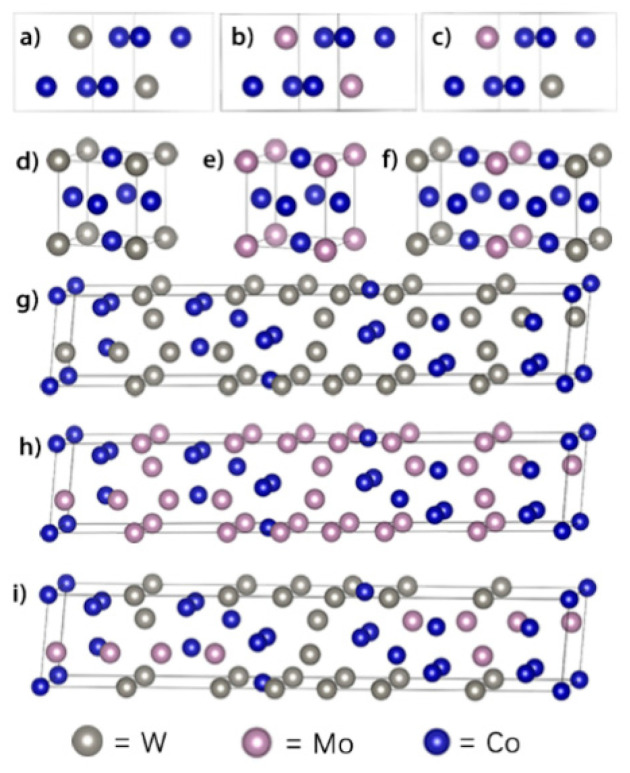
The crystalline structures of (**a**) Co_3_W_DO_19_; (**b**) Co_3_Mo_DO_19_; (**c**) Co_6_WMo_DO_19_; (**d**) Co_3_W_L1_2_; (**e**) Co_3_Mo_L1_2_; (**f**) Co_6_WMo_L1_2_; (**g**) Co_7_W_6__μ; (**h**) Co_7_Mo_6__μ; and (**i**) Co_7_W_3_Mo_3__μ.

In this study, nine intermetallic compounds in the Co–W, Co–Mo, and Co–W–Mo alloys have been studied, i.e., Co_3_W_DO_19_ (P6_3_/mmc), Co_3_Mo_DO_19_ (P6_3_/mmc), Co_3_W(Mo) _DO_19_ (P6_3_/mmc), Co_3_W_L1_2_ (Fm-3m), Co_3_Mo_L1_2_ (Fm-3m), Co_3_W(Mo)_L_12_ (Fm-3m), Co_7_Mo_6__μ(R-3m), Co_7_W_6__μ (R-3m) and Co_7_W(Mo)_6__μ (R-3m). The structural models of the nine alloys are presented in Figure 1. The calculated ground-state lattice parameters *a* and *c* of the DO_19_, L1_2_ and μ phases of the binary and ternary intermetallic compounds are listed in Table 1 (*a* = *b*; thus, *b* is omitted here) and the site occupation in structures are attached in the Supplementary Materials. In addition, the parameters have been compared with the available experimental data and other theoretical results. As shown in Table 1, the calculated lattice parameters are consistent with the experimental values, with the average deviation less than 1.0%. Subsequently, the cohesive energy (Δ*E*) and formation energy (Δ*H*) have been calculated to investigate the chemical stability of the compounds, as shown in Table 1. The Δ*E* and Δ*H* values have been defined by Equations (4) and (5), respectively.

(4) $$∆E\left(A_{x}B_{y}\right)=\frac{\left[E_{total}\left(A_{x}B_{y}\right)-xE_{atom}\left(A\right)-yE_{atom}\left(B\right)\right]}{x+y}$$

(5) $$∆H\left(A_{x}B_{y}\right)=\frac{\left[E_{total}\left(A_{x}B_{y}\right)-xE_{solid}\left(A\right)-yE_{solid}\left(B\right)\right]}{x+y}$$

where *x*, *y*, *E_total_*, *E_atom_* and *E_solid_* represent the content of element A in the unit cell, content of element B in the unit cell, total energy of the unit cell, energy of the isolated atom and ground state energy of the pure metal, respectively. Generally, Δ*H* can be used to characterize the phase stability of the intermetallic compounds. The more negative the Δ*H* value, the higher the phase stability. Due to the calculations carried out at 0 K in first-principles calculations, the results should be effective in low temperature. It can be observed from Table 1 that the formation energy of DO_19_-ordered Co_3_X is more negative than L1_2_-ordered Co_3_X, thus implying that DO_19_-Co_3_X is more stable at low temperature. This observation is consistent with the experimental data and other theoretical results. In fact, the L1_2_-ordered Co_3_X is detected in Co–W(Al) systems, but it is difficult to be prepared and found in the pure binary Co–W system. Compared with DO_19_-Co_3_X, μ-ordered Co_7_X_6_ displays a lower stability. According to the experimental results, the alloying processes of Co–W/Mo obey the orders from $Co+X\to Co_{3}X\to Co_{7}X_{6}$ (X = W or Mo) with the increasing temperature, as depicted in the reactions of Equations (6) and (7). The inference, therefore, is that $Co_{7}X_{6}$ should be more stable at high temperature.

(6) $$3Co+X=Co_{3}X$$

(7) $$7Co_{3}X+18X=3Co_{7}X_{6}$$

### 3.2. Elastic Properties

Elastic stiffness (*C_ij_*) and compliance (*S_ij_*) tensors were used to describe the response of the crystal to the external stress applied in different directions. These are useful for understanding the mechanical and physical properties of the alloys, such as machinability, bonding characteristics, and ductility. There were nine independent elastic stiffness constants for the anisotropic crystal: *C*_11_, *C*_12_, *C*_13_, *C*_22_, *C*_23_, *C*_33_, *C*_44_, *C*_55_ and *C*_66_. In addition, the *S_ij_* quantities represent the inverse matrix of *C*_ij_, as defined in Equation (8):

(8) $$S_{ij}=C_{ij}{}^{-1}$$

Due to the crystal symmetry, three independent elastic constants (i.e., *C*_11_, *C*_12_ and *C*_44_) exist for the L1_2_-ordered cubic structure, and Equation (8) can be simplified as:

(9) $$(\begin{array}{c} \sigma_{1}\\ \sigma_{2}\\ \sigma_{3}\\ \sigma_{4}\\ \sigma_{5}\\ \sigma_{6} \end{array})=(\begin{array}{ccc} \begin{array}{c} \begin{array}{cc} C_{11} & \;C_{12} \end{array}\\ \begin{array}{cc} C_{12} & \;C_{11} \end{array} \end{array} & \begin{array}{c} \begin{array}{cc} C_{12} & \;0 \end{array}\\ \begin{array}{cc} C_{12} & \;0 \end{array} \end{array} & \begin{array}{c} \begin{array}{cc} 0 & \;0 \end{array}\\ \begin{array}{cc} 0 & \;0 \end{array} \end{array}\\ \begin{array}{c} \begin{array}{cc} C_{12} & \;C_{12} \end{array}\\ \begin{array}{cc} 0 & \;0 \end{array} \end{array} & \begin{array}{c} \begin{array}{cc} C_{11} & \;0 \end{array}\\ \begin{array}{cc} \;0 & \;C_{44} \end{array} \end{array} & \begin{array}{c} \begin{array}{cc} 0 & \;0 \end{array}\\ \begin{array}{cc} 0 & \;0 \end{array} \end{array}\\ \begin{array}{c} \begin{array}{cc} 0 & \;0 \end{array}\\ \begin{array}{cc} 0 & \;0 \end{array} \end{array} & \begin{array}{c} \begin{array}{cc} 0 & \;0 \end{array}\\ \begin{array}{cc} 0 & \;0 \end{array} \end{array} & \begin{array}{c} \begin{array}{cc} C_{44} & 0 \end{array}\\ \begin{array}{cc} 0 & \;C_{44} \end{array} \end{array} \end{array})(\begin{array}{c} e_{1}\\ e_{2}\\ e_{3}\\ e_{4}\\ e_{5}\\ e_{6} \end{array})$$

For the DO_19_-ordered hexagonal structure and μ phase, two more independent elastic constants (i.e., *C*_13_ and *C*_33_) can be added, and Equation (8) can be expressed as:

(10) $$(\begin{array}{c} \sigma_{1}\\ \sigma_{2}\\ \sigma_{3}\\ \sigma_{4}\\ \sigma_{5}\\ \sigma_{6} \end{array})=(\begin{array}{cccc} \begin{array}{c} \begin{array}{cc} C_{11} & \;C_{12} \end{array}\\ \begin{array}{cc} C_{12} & \;C_{11} \end{array} \end{array} & \begin{array}{c} \begin{array}{cc} C_{13} & \;0 \end{array}\\ \begin{array}{cc} C_{13} & \;0 \end{array} \end{array} & \begin{array}{c} 0\\ 0 \end{array} & \begin{array}{c} 0\\ 0 \end{array}\\ \begin{array}{c} \begin{array}{cc} C_{13} & \;C_{13} \end{array}\\ \begin{array}{cc} 0 & \;0 \end{array} \end{array} & \begin{array}{c} \begin{array}{cc} C_{33} & \;0 \end{array}\\ \begin{array}{cc} 0 & \;C_{44} \end{array} \end{array} & \begin{array}{c} 0\\ 0 \end{array} & \begin{array}{c} 0\\ 0 \end{array}\\ \begin{array}{c} \begin{array}{cc} 0 & \;0 \end{array}\\ \begin{array}{cc} 0 & \;0 \end{array} \end{array} & \begin{array}{c} \begin{array}{cc} 0 & \;0 \end{array}\\ \begin{array}{cc} 0 & \;0 \end{array} \end{array} & \begin{array}{c} C_{44}\\ 0 \end{array} & \begin{array}{c} 0\\ \frac{C_{11}-C_{12}}{2} \end{array} \end{array})\left(\begin{array}{c} e_{1}\\ e_{2}\\ e_{3}\\ e_{4}\\ e_{5}\\ e_{6} \end{array}\right)$$

The mechanical stability of the Co–W–Mo alloy can be determined from the calculated elastic constants. For the cubic L1_2_-Co_3_X crystals, the mechanical stability criteria can be written as [33]:

(11) $$C_{11}>0$$

(12) $$C_{44}>0$$

(13) $$C_{11}-C_{12}>0$$

(14) $$C_{11}+2C_{12}>0$$

The mechanical stability criteria for the DO_19_-Co_3_X and μ-Co_7_X_6_ structures can be expressed as follows [34]:

(15) $$C_{11}-C_{12}>0$$

(16) $$C_{11}+C_{33}-2C_{13}>0$$

(17) $$2C_{11}+C_{33}+2C_{12}+4C_{13}>0$$

(18) $$C_{11}+2C_{12}>0$$

The *C_ij_* and *S_ij_* values of the Co_3_X and Co_7_X_6_ compounds have been calculated at 0 K and are tabulated in Table 2. The elastic constants obtained in this study were observed to be in good agreement with the previously reported results [15]. It should be noted that L1_2_- and DO_19_-Co_3_X and Co_7_X_6_ are mechanically stable at 0 K, as per Equations (11)–(15).

The bulk and shear moduli are used to evaluate the resistance to the volume change and reversible deformation at a given stress, and the Young’s modulus is applied to estimate the stiffness of materials. The Poisson’s ratio is associated with the resistance of the material against shear. Based on the Voigt and Reuss theories [35], the bulk and shear moduli can be calculated by using Equations (19)–(24):

(19) $$9B_{v}=\left(C_{11}+C_{22}+C_{33}\right)+2\left(C_{12}+C_{23}+C_{31}\right)$$

(20) $$15G_{v}=\left(C_{11}+C_{22}+C_{33}\right)-\left(C_{12}+C_{23}+C_{31}\right)+3\left(C_{44}+C_{55}+C_{66}\right)$$

(21) $$1/B_{R}=\left(S_{11}+S_{22}+S_{33}\right)+2\left(S_{12}+S_{23}+S_{31}\right)$$

(22) $$15/G_{R}=\left(S_{11}+S_{22}+S_{33}\right)-4\left(S_{12}+S_{23}+S_{31}\right)+3\left(S_{44}+S_{55}+S_{66}\right)$$

(23) $$B=\left(B_{v}+B_{R}\right)/2$$

(24) $$G=\left(G_{v}+G_{R}\right)/2$$

where *V* and *R* denote the values calculated by using the Voigt and Reuss theories, respectively.

On the basis of the elastic constants, the bulk, shear and Young’s moduli as well as Poisson’s ratio at the ground state can be calculated by using the Voigte–Reuss–Hill method:

(25) $$\sigma =\frac{1}{2}\left[1-\frac{3G}{3B+G}\right],\;\;\frac{1}{E}=\frac{1}{3G}+\frac{1}{9B}$$

**Figure 2:**
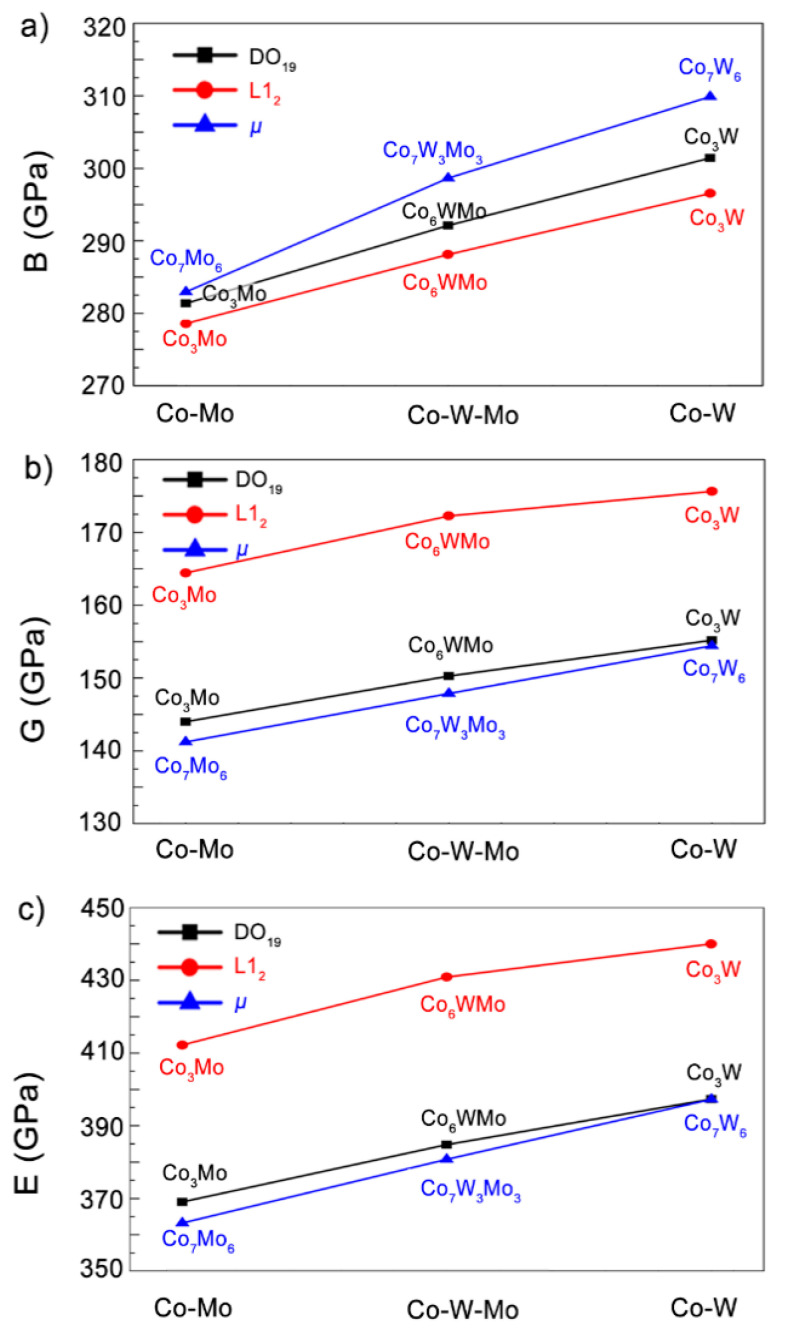
The comparisons of the (**a**) bulk (*B*), (**b**) shear (*G*) and (**c**) Young’s (*E*) moduli of the Co–W/Mo compounds in the DO_19_, L1_2_ and μ structures.

The bulk modulus measures the resistance offered by the material against the changes in its volume. From Figure 2a, the μ-Co_7_X_6_ phase is observed to exhibit the highest bulk modulus, whereas L1_2_-Co_3_X possesses the lowest bulk modulus in the system with the same X element (X = Mo or W). The results also indicate that Co–W has a higher bulk modulus than Co–M and Co–W–Mo possessing the same structures, thus demonstrating the superior strength of the Co–W alloys as compared to Co–Mo. The Young’s modulus is an effective indicator of the stiffness of the material. The higher the Young’s modulus, the stiffer the material. As observed in Figure 2c, the Young’s modulus decreases in the following sequence: L1_2_ > DO_19_ > μ, thus indicating that L1_2_-Co_3_X is stiffer than the other structures, which corresponds with the experimental findings that the L1_2_ phase exists as a precipitation strengthening compound in the Co-based alloys. 

The brittleness and ductility of Co–W/Mo have been studied by taking the ratio of the bulk modulus to the shear modulus (*B*/*G*) [36]. A high *B*/*G* value indicates an optimal ductility, whereas a low *B*/*G* value suggests the material brittleness. The critical borderline value between the ductile and brittle materials has been evaluated to be 1.75. The Poisson’s ratio is employed to estimate the stability of a material against the shear deformation and usually ranges from −1 to 0.5. A large Poisson’s ratio represents an effective plasticity. Basically, the material is considered to be ductile in cases where *σ* is higher than 0.26. Hence, based on the obtained *B*/*G* ratio for Co–W/Mo in Table 2, DO_19_-Co_3_X and μ-Co_7_X_6_ possess a ductile character, and L1_2_-Co_3_X represents more brittle materials. Moreover, the value of the Poisson’s ratio stands for the degree of directionality of the covalent bonds. For the ionic crystals, the *σ* value is usually close to 0.25, whereas the *σ* value is around 0.1 for the covalent materials [37]. As can be observed from Table 2, the computed values of the Poisson’s ratio are greater than 0.25. Therefore, the ionic bond interactions significantly contribute to the interatomic bonding in the Co–W–Mo binary and ternary compounds.

In comparison with the previous study results of the Ni-based intermetallic phase, the bulk, shear, and Young’s moduli of Co–W/Mo in this work and the previous works were all higher than the Ni-based alloys such as Ni_2_Mo_2_, Ni_2_Mo, Ni_3_Mo, Ni_4_W, Ni_3_W, etc. However, the *B*/*G* of Ni-based alloys are usually higher than Co–W/Mo alloys [38,39,40]. This means that the Co–W/Mo would perform better on strength but worse on ductility tests than Ni-based alloy. This may be caused by the stronger Co3d–W5p/Mo4p orbital hybridization interaction between Co and W/Mo atoms than Ni3d–W5p/Mo4p, because the difference of electronegativity between Ni–W/Mo is smaller than Co–W/Mo. 

Comparing the isomorphous compounds with different elements, the Co–W alloys exhibit higher strength than the Co–Mo alloys, whereas the strength of the Co–W–Mo alloys is located between them. The similar structures and properties of the Co–W and Co–Mo alloys enable them to exhibit the continuous solid solution phase-like behavior of the tungsten and molybdenum alloys. Therefore, the method involving the continuous adjustment of the mechanical properties leads to an effective design of the Co–W/Mo alloy. However, it is worth noting that the mechanical properties of the Co_6_WMo phases are closer to the Co–W alloy instead of the Co–Mo alloy. The W atoms have a larger radius than the Mo atoms; thus, the W atoms in the ternary alloys would play a vital role during the deformation process.

### 3.3. Thermodynamic Properties

In this section, the quasi-harmonic Debye model has been applied to investigate the thermodynamic properties of the Co–W/Mo compounds in the L1_2_, DO_19_ and μ structures at a finite temperature [41]. The non-equilibrium Gibbs energy of the crystal phase at fixed temperature and hydrostatic pressure can be expressed as:

(26) $$G^{*}\left(V;p,T\right)=E\left(V\right)+\mathrm{pV}+A_{vib}\left[\Theta \left(V\right);T\right]$$

where *E*(*V*) is the total energy of the crystal in a given volume, *A_vib_* is the Helmholtz vibrational free energy, and Θ is the Debye temperature. Adopting the Debye model of the phonon density of states, the vibrational contribution of *A_vib_* can be written as [42,43]:

(27) $$A_{vib}\left(\Theta ;T\right)=nkT\left[\frac{9}{8}\frac{\Theta }{T}+3ln\left(1-e^{-\frac{\Theta }{T}}\right)-D\left(\frac{\Theta }{T}\right)\right]$$

where *n* is the number of atoms per formula unit, and *k* is the Boltzmann constant. D(*y*) is the Debye integral, which is defined as:

(28) $$D\left(y\right)=\frac{3}{y^{3}}\overset{y}{\underset{0}{\int }}\frac{x^{3}}{e^{x}-1}dx$$

The Debye temperature Θ, related to the average sound velocity, can be calculated as:

(29) $$\Theta =\frac{\hbar }{k}{\left[6\pi^{2}V^{1/2}n\right]}^{1/3}f\left(v\right)\sqrt{\frac{B_{s}}{M}})$$

where *M* refers to the molecular mass per unit cell, and *k* is the reduced Planck constant. *B_s_* is the adiabatic bulk modulus, approximated by using the static compressibility, and can be written as [44,45]:

(30) $$B_{s}\approx B\left(V\right)=V\left(\frac{d^{2}E\left(V\right)}{dV^{2}}\right)$$

Hence, the (*p*, *T*) thermal equilibrium can be obtained by solving the non-equilibrium Gibbs function, given as:

(31) $${\left(\frac{\partial G^{*}\left(V;p,T\right)}{\partial V}\right)}_{p,T}=0$$

For the equilibrium state at a given (*p*, *T*), the thermodynamic properties such as isochoric heat capacity (*C_v_*), isobaric heat capacity (*C_p_*), vibrational entropy (*S_vib_*) and volume thermal expansion coefficient (α) can be calculated by using the following equations:

(32) $$C_{v}=3nk\left[4D\left(\frac{\Theta }{T}\right)-\frac{3\Theta /T}{e^{\Theta /T}-1}\right]$$

(33) $$C_{p}=C_{v}\left(1+\alpha \gamma T\right)$$

(34) $$S_{vib}=3nk\left[\frac{4}{3}D\left(\frac{\Theta }{T}\right)-ln\left(1-e^{-\frac{\Theta }{T}}\right)\right]$$

(35) $$\alpha =\frac{\gamma C_{v}}{B_{T}V},\;\;\;B_{T}\left(p,T\right)=-V{\left(\frac{\partial p}{\partial V}\right)}_{T}$$

where *B_T_* is the isothermal bulk modulus, and the Grüneisen parameter γ is defined as:

(36) $$\gamma =-\frac{dln\Theta \left(V\right)}{dlnV}$$

**Figure 3:**
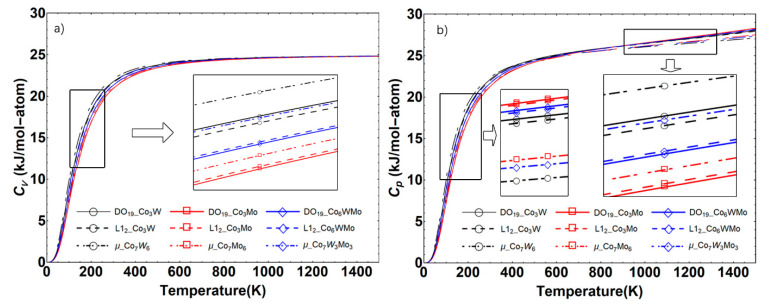
(**a**) Isochoric heat capacity (*C_v_*) and (**b**) isobaric heat capacity (*C_p_*) of the Co–W/Mo compounds in the DO_19_, L1_2_ and μ structures as a function of temperature.

The calculated *C_v_* and *C_p_* values of the Co–W/Mo compounds in the L1_2_, DO_19_ and μ structures at 0 GPa are illustrated in Figure 3. Herein, the thermodynamic properties have been calculated by ignoring the thermal electronic contribution and effect of the zero-point energy.

As can be observed from Figure 3a, the *C_v_* values of the phases increase rapidly at sufficiently low temperatures, which coincides with the Debye model theory. As the temperature reaches 900 K, the *C_v_* values become almost constant (25 J/K mol), which is known as the Dulonge Petit limit [46]. In Figure 3b, the *C_p_* values of the compounds are noted to be proportional to T^3^ at temperatures near 0 K. Furthermore, the *C_p_* values of the Co–W phases are higher than Co–W–Mo and Co–Mo, which is consistent with the *C_p_* value of W being larger than Mo at 300 K. Co_7_X_6_ and DO_19_-Co_3_X exhibited the lowest and highest *C_p_* values at high temperatures, respectively (shown in Figure 3b). The observed phenomenon can be associated with the stability and melting points of the different phases at high temperatures, and Co_7_W_6_ generally has the highest melting points in these phases due to the high melting point of W.

**Figure 4:**
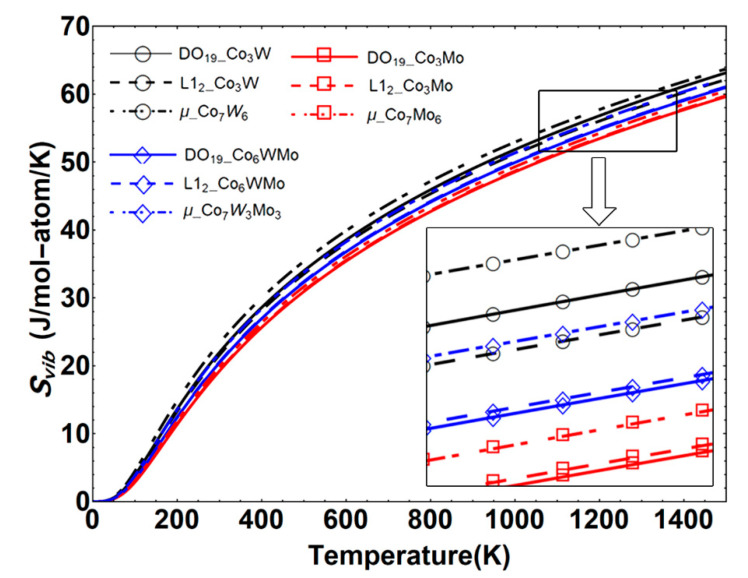
The enthalpies of the Co–W/Mo compounds in the DO_19_, L1_2_ and μ structures as a function of temperature.

As is well known, the enthalpy, as a function of temperature, is a crucial parameter in thermodynamic modeling. The calculated enthalpies of the Co_3_X compounds in the DO_19_, L1_2_ and μ structures at zero pressure are shown in Figure 4. Similar to the heat capacity, the findings are almost identical with the order of enthalpy of Co, W and Mo reported earlier [47] (the enthalpy of W is higher than that of Mo in the range 300–1500 K).

**Figure 5:**
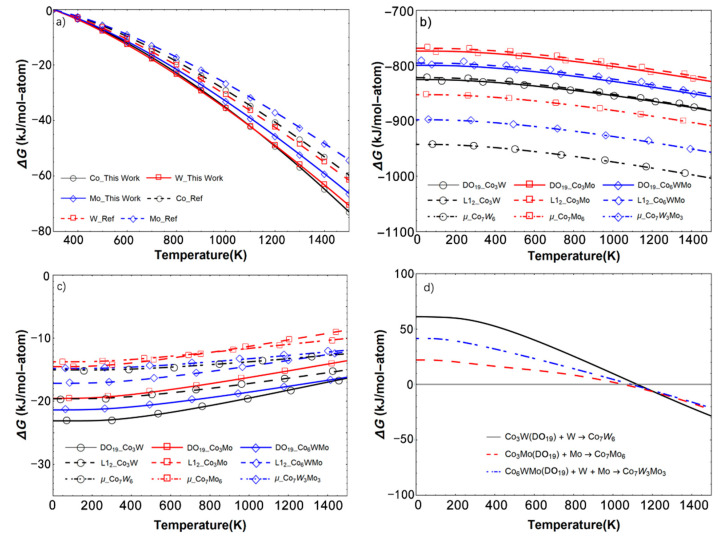
(**a**) The calculated and recommended Δ*G* of Co, W and Mo (the values at 300 K represent the benchmark); (**b**) the calculated mean values of the Co–W/Mo compounds in the DO_19_, L1_2_ and μ structures; (**c**) formation Gibbs free energy of the Co–W/Mo compounds in the DO_19_, L1_2_ and μ structures; and (**d**) Δ*G* of the phase transformation from Co_3_X to Co_7_X_6_.

The calculated Gibbs free energy of DO_19_-, L1_2_- and μ-ordered Co–W/Mo are shown in Figure 5. In order to verify the reliability of the obtained values, the calculated and experimental Gibbs free energy [47] values of pure solid Co, Mo and W from 300 K to 1500 K are displayed in Figure 5a. Herein, the Gibbs free energy of the pure elements at 300 K was set as the benchmark. The calculated Δ*G* values were noted to be smaller than the experimental values, which can be associated with the contribution of the thermal electrons to entropy (*S*) at high temperatures. However, the atoms are conserved before and after the alloying process, and the *S* of the thermal electrons is primarily determined by the atoms. The relative errors generated from the thermal electrons to Δ*S* of the alloying reaction at a specific temperature should be smaller than the errors originating from enhancing the temperature. Figure 5b presents the calculated Gibbs free energy of the DO_19_-, L1_2_-, and μ-ordered Co–W/Mo compounds as a function of temperature. The Co–W phases are noted to have a lower Gibbs free energy in comparison with the Co–Mo and Co–W–Mo alloys, in accordance with the findings for the pure elements. 

In order to effectively analyze the stability of the phases at different temperatures, the formation Gibbs free energy Δ*G*_f_ has been calculated using Equation (37) and is shown in Figure 5c:

(37) $$∆G_{f}\left(A_{x}B_{y}\right)\;=\;\frac{\left[G_{total}\left(A_{x}B_{y}\right)-xG_{solid}\left(A\right)-yG_{solid}\left(B\right)\right]}{x+y}$$

where *G*_total_ (*A*_x_ *B*_y_), *G*_solid_ (*A*) and *G*_solid_ (*B*) are the Gibbs free energy values of the unit cell of *A*_x_ *B*_y_, *A* and *B*, respectively. As shown in Figure 5c, the Δ*G*_f_ values are significantly related to the crystal structure, and the DO_19_-Co_3_X phases have the lowest Δ*G*_f_ as compared with the other two phases possessing the same elements. However, it can be noticed that the Δ*G*_f_ difference between the DO_19_ and μ structures is inversely proportional to the increasing temperature. According to the phase transformation from DO_19_-Co_3_X to Co_7_X_6_ in the experiments. The Δ*G*_f_ of the follow reactions (38–40) are calculated and shown in Figure 5d:

(38) $$Co_{3}W+W\to Co_{7}W_{6}$$

(39) $$Co_{3}Mo+Mo\to Co_{7}Mo_{6}$$

(40) $$Co_{6}WMo+W+Mo\to Co_{7}W_{3}Mo_{3}$$

As can be observed from Figure 5d, Co_3_X and X react to form Co_7_X_6_ in the temperature range 1000–1200 K, and the transformation temperature of Co–Mo from Co_3_Mo to Co_7_Mo_6_ is noted to be lower than that of Co–W by about 100 K. Comparing the Co–X binary diagram, DO_19_-Co_3_Mo and Co_3_W disappear after heating at about 1050 °C and 1100 °C, respectively. Hence, the phase transformation temperature of Co_3_Mo was lower than Co_6_WMo and Co_3_W in both experimental and theoretical analyses. In addition, the Δ*G* of the Co–W system for the phase transformation from Co_3_W to Co_7_W_6_ was higher than the Co–Mo system. It indicates the high stability of Co_7_W_6_ at high temperatures, which is consistent with the previously observed improvement in the stability of the μ-phase of Co_7_Mo_6_ on incorporating tungsten.

## 4. Summary

The DO_19,_ L1_2_ and μ structural phases in the Co–W, Co–Mo and Co–W–Mo compounds have been considered in this study for calculating the mechanical and thermodynamic properties. Possessing the same elements, the DO_19_- and μ-phases exhibited a lower shear modulus and higher ductility than the L1_2_-phases. On the other hand, with the same structure and different elements, Co–W displayed higher strength than Co–W–Mo and Co–Mo. Moreover, the mechanical properties of Co–W–Mo with the same amount of W and Mo were noted to be closer to Co–W than Co–Mo. The assessment presented in this study reveals the lack of the experimental data with respect to the thermodynamic properties of Co–W/Mo. The *C_v_* and *C_p_* values of the nine phases are noted to match well with the regular pattern of the elements obtained experimentally. Coinciding with the phase transformation, DO_19_-Co_3_X transforms to Co_7_X_6_ at high temperatures. Besides, the properties of the Co–W–Mo ternary alloys are noted to hover between the Co–W and Co–Mo binary systems. The findings obtained in this study are expected to provide valuable clues for the design of novel Co-based materials and can be helpful in stimulating future experimental and theoretical research in the field of Co–W/Mo alloys.

## Figures and Tables

**Table 1:** The calculated equilibrium structural parameters (structure and lattice parameters a and c in Å) compared with the available experimental data [30,31,32], along with the cohesive (ΔE) and formation (ΔH) energies of the Co3X (DO19 and L12) and Co7X6 compounds (in eV per atom).

Compound	Structure	Calculated (Å)		Experimental (Å)		Δ*E*	Δ*H*
		*a*	*b*	*c*	*d*	(eV/atom)	(eV/atom)
Co_3_W	DO_19_	5.120	4.115	5.120 [30]	4.116 [30]	−7.682	−0.238
Co_3_W	L1_2_	3.590	3.590			−7.640	−0.195
Co_3_Mo	DO_19_	5.097	4.076	5.125 [31]	4.113 [31]	−7.486	−0.196
Co_3_Mo	L1_2_	3.585	3.585			−7.427	−0.138
Co_6_WMo	DO_19_	5.101	4.082			−7.585	−0.218
Co_6_WMo	L1_2_	3.589				−7.533	−0.166
Co_7_W_6_	μ	4.743	25.590	4.751 [30]	25.617 [30]	−8.554	−0.146
Co_7_Mo_6_	μ	4.737	25.417	4.762 [32]	25.617 [32]	−8.219	−0.096
Co_7_W_3_Mo_3_	μ	4.745	25.476			−8.399	−0.134

**Table 2:** The calculated elastic properties of the Co_3_X (DO_19_ and L1_2_) and Co_7_X_6_ compounds.

Structures	Co_3_W	Co_3_Mo	Co_6_WMo	Co_3_W	Co_3_Mo	Co_6_WMo	Co_7_W_6_	Co_7_Mo_6_	Co_7_W_3_Mo_3_
	DO_19_	DO_19_	DO_19_	L1_2_	L1_2_	L1_2_	μ	μ	μ
*C*_11_(GPa)	501.463	462.259	484.533	430.166	408.132	418.138	516.285	472.228	490.915
*C*_22_(GPa)	501.463	462.259	484.533	430.166	408.132	418.138	516.285	472.228	490.915
*C*_33_(GPa)	539.047	505.988	522.428	430.166	408.132	418.635	571.091	504.111	537.801
*C*_44_(GPa)	116.850	109.096	113.547	185.551	170.768	182.684	110.768	102.862	107.542
*C*_55_(GPa)	116.850	109.096	113.547	185.551	170.768	182.684	110.768	102.862	107.542
*C*_66_(GPa)	144.633	134.472	139.718	185.551	170.768	182.256	147.279	135.152	143.475
*C*_12_(GPa)	212.196	198.316	205.097	229.722	213.797	223.863	225.810	203.879	210.551
*C*_13_(GPa)	186.658	176.421	181.877	229.722	213.797	222.571	183.424	172.516	186.904
*C*_23_(GPa)	186.658	176.421	181.877	229.722	213.797	222.569	183.424	172.516	186.904
*S*_11_(MPa)	2.584	2.824	2.677	3.701	3.829	3.807	2.522	2.759	2.658
*S*_22_(MPa)	2.584	2.824	2.677	3.701	3.829	3.807	2.522	2.759	2.658
*S*_33_(MPa)	2.265	2.429	2.345	3.701	3.829	3.783	2.082	2.403	2.282
*S*_44_(MPa)	8.558	9.166	8.807	5.389	5.856	5.474	9.028	9.722	9.299
*S*_55_(MPa)	8.558	9.166	8.807	5.389	5.856	5.474	9.028	9.722	9.299
*S*_66_(MPa)	6.914	7.436	7.157	5.389	5.856	5.487	6.790	7.399	6.970
*S*_12_(MPa)	−0.873	−0.964	−0.901	−1.288	−1.316	−1.340	−0.920	−0.967	−0.909
*S*_13_(MPa)	−0.593	−0.649	−0.618	−1.288	−1.316	−1.312	−0.514	−0.613	−0.608
*S*_23_(MPa)	−0.593	−0.649	−0.618	−1.288	−1.316	−1.312	−0.514	−0.613	−0.608
*B*(GPa)	301.429	281.370	292.109	296.537	278.575	288.102	309.871	282.932	298.647
*G*(GPa)	155.180	144.002	150.249	175.623	164.428	172.261	154.418	141.217	147.852
*E*(GPa)	397.352	369.047	384.777	440.004	412.187	430.902	397.264	363.220	380.726
*σ*	0.280	0.281	0.280	0.253	0.253	0.251	0.286	0.286	0.288
*B*/*G*	1.942	1.954	1.944	1.688	1.694	1.672	2.007	2.004	2.020

## Data Availability

The data presented in this study are available in the article and supplementary material.

## Supplementary Materials

The following are available online at https://www.mdpi.com/1996-1944/14/6/1404/s1.
